# Psychological outcomes, knowledge and preferences of pregnant women on first-trimester screening for fetal structural abnormalities: A prospective cohort study

**DOI:** 10.1371/journal.pone.0245938

**Published:** 2021-01-27

**Authors:** Francesca Bardi, Merel Bakker, Monique J. A. Kenkhuis, Adelita V. Ranchor, Marian K. Bakker, Ayten Elvan-Taşpınar, Erwin Birnie, Caterina M. Bilardo

**Affiliations:** 1 Department of Obstetrics and Gynecology, University Medical Center Groningen, University of Groningen, Groningen, The Netherlands; 2 Department of Health Psychology, University Medical Center Groningen, University of Groningen, Groningen, The Netherlands; 3 Department of Genetics, University Medical Center Groningen, University of Groningen, Groningen, The Netherlands; 4 Department of Obstetrics and Gynecology, Amsterdam University Medical Centers, Amsterdam, The Netherlands; University of Mississippi Medical Center, UNITED STATES

## Abstract

**Introduction:**

The primary aim of this study is to investigate the impact of a 13-week anomaly scan on the experienced levels of maternal anxiety and well-being. Secondly, to explore women’s knowledge on the possibilities and limitations of the scan and the preferred timing of screening for structural abnormalities.

**Material and methods:**

In a prospective-cohort study conducted between 2013–2015, pregnant women in the North-Netherlands underwent a 13-week anomaly scan. Four online-questionnaires (Q1, Q2, Q3 and Q4) were completed before and after the 13- and the 20-week anomaly scans. In total, 1512 women consented to participate in the study and 1118 (74%) completed the questionnaires at Q1, 941 (64%) at Q2, 807 (55%) at Q3 and 535 (37%) at Q4. Psychological outcomes were measured by the state-trait inventory-scale (STAI), the patient’s positive-negative affect (PANAS) and ad-hoc designed questionnaires.

**Results:**

Nine-nine percent of women wished to be informed as early as possible in pregnancy about the absence/presence of structural abnormalities. In 87% of women levels of knowledge on the goals and limitations of the 13-week anomaly scan were moderate-to-high. In women with a normal 13-week scan result, anxiety levels decreased (*P* < .001) and well-being increased over time (*P* < .001). In women with false-positive results (n = 26), anxiety levels initially increased (STAI-Q1: 39.8 vs. STAI-Q2: 48.6, *P* = 0.025), but later decreased around the 20-week anomaly scan (STAI-Q3: 36.4 vs. STAI-Q4: 34.2, *P* = 0.36).

**Conclusions:**

The 13-week scan did not negatively impact the psychological well-being of pregnant women. The small number of women with screen-positive results temporarily experienced higher anxiety after the scan but, in false-positive cases, anxiety levels normalized again when the abnormality was not confirmed at follow-up scans. Finally, most pregnant women have moderate-to-high levels of knowledge and strongly prefer early screening for fetal structural abnormalities.

## Introduction

In current clinical practice, prenatal screening programs target both chromosomal and structural abnormalities, with paradigms varying among countries [1]. Screening for structural abnormalities is routinely performed by ultrasound between 18–22 weeks of gestation. However, increasing evidence has suggested that about half of fetal structural abnormalities can be detected during the first trimester of pregnancy with low false-positive rates [2, 3]. Especially lethal defects, reasons for parents to opt for termination of pregnancy (TOP) [4], are amenable to early diagnosis [2, 3, 5, 6]. Therefore, early detection of structural abnormalities has two main advantages. First, it offers more time for additional ultrasound and genetic investigations and second, it allows parents to make an unrushed and well-informed decision regarding the continuation or termination of pregnancy, well within the legal limit for TOP. This is especially relevant as early TOP is associated with less psychological sequelae for the mother [7]. With the growing number of available prenatal tests, it is even more challenging for healthcare providers to counsel pregnant women and their partners on the various combinations of options. Prerequisite for making an informed decision is that women fully understand the coverage and limitations of the different prenatal screening tests. Most studies evaluating the performance of a 13-week scan focus on detection rates, screen-positive rates and positive predictive value, with little attention to the psychological outcomes of screening on women. Therefore, aspects that still need consideration are, next to the preferences of pregnant women, the psychological outcomes of test-negative and test-positive screening results [8]. The aim of this study is to investigate the psychological outcomes of a 13-week anomaly scan in pregnant women who were offered the scan in a study setting. Specifically, we explore the differences in levels of experienced anxiety and maternal well-being induced by the scan. Secondly, we investigate women’s preferences regarding the timing of screening and their knowledge on the goals and limitations of the 13-week anomaly scan.

## Materials and methods

### Prenatal screening program

Since 2007, in the Netherlands, every pregnant woman is counseled regarding the available options for prenatal screening. Screening for chromosomal abnormalities is currently done by cell-free DNA testing or by the combined test (CT). Screening for structural abnormalities is done at 18–21 weeks by an ultrasound performed by certified sonographers according to an established protocol [9].

### Study design and participants

**Fig 1:**
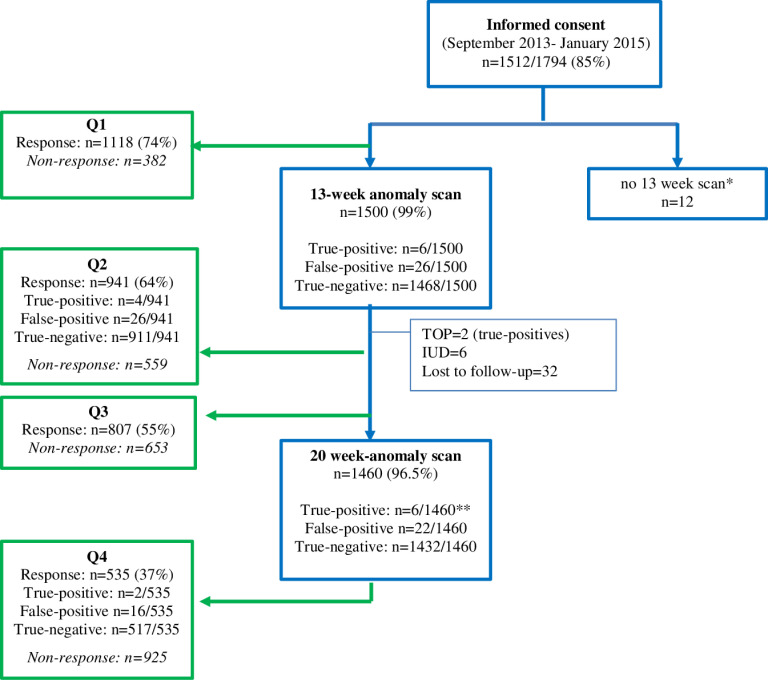
Study profile. *Fetal loss, lost to follow-up. **The 6 abnormalities detected at the 20-week anomaly scans different from those found at the 13-week anomaly scans. Blue: women who initially consented to participate. Green: women who filled in the questionnaires.

Between September 2012-December 2015, women in the Northern-Netherlands who opted for the CT, were invited to participate in a multicenter-prospective cohort study [2], aimed at investigating the yield of a systematic examination of fetal anatomy at the time of the CT (11–13 weeks). Women participating in the study between September 2013-January 2015 (n = 1794) were also invited to enroll in the sub-study aimed at investigating their psychological outcomes. In total, 1512/1794 (85%) women consented to participation in the psychological study (Fig 1). All women received written information on the study by their midwives and signed an informed-consent form. Participating centers were three first-line ultrasound practices, one second-line medical center and one third-line academic center. Women were also offered a 20-week anomaly scan according to the national prenatal screening program. Women below 18 years of age or unable to understand the Dutch language were excluded. For the study, a license was obtained from the Ministry of Health within the Dutch Population Screening Act11, regulating screening for incurable diseases [10]. No funding was available for this study.

### Data collection

Questionnaire answers were collected electronically before and after the 13-week anomaly scan (Q1, Q2) and before and after the 20-week anomaly scan (Q3, Q4). Questionnaires were written in Dutch and made available online by Unipark Online-Umfrage software. All answers were automatically entered in an electronic database. The questionnaire content is illustrated below.

### Maternal characteristics

Maternal socio-demographic characteristics (age, marital/household status, religious background, educational/employment status) and obstetric history (parity, previous miscarriages/TOPs and (family) history of congenital abnormalities) were recorded at study entry.

### Psychological outcomes

#### Anxiety (Q1-Q4).

The validated 6-item short version of the state-trait anxiety inventory scale (STAI, Dutch version) [11] was used to measure levels of experienced anxiety before and after the scans (Q1-Q4). Response modality was a four-point Likert scale (1 = not at all, 4 = very much). The prorated STAI-score was obtained by STAI-6 scores multiplied by 20 and divided by 6 [11]. Score-range was 20–80, with anxiety increasing at higher scores. Internal reliability was good at Q1-Q4 with Cronbach’s-alpha ranging from 0.83–0.86. STAI-scores of 40 or higher were considered clinically relevant levels of anxiety [12].

#### Affect (Q1-Q4).

The validated positive-negative affect schedule (PANAS, Dutch version, score range 10–50 for each scale) [13] was used to assess affect. The PANAS consists of two 10-item scales measuring positive-affect (PA: active, alert, attentive, determined, enthusiastic, excited, inspired, interested, proud, strong) and negative-affect (NA: ashamed, distressed, guilty, hostile, jittery, nervous, scared, upset). Participants were asked to indicate how they felt on a five-point Likert scale (1 = ‘not at all’, 5 = ‘extremely’). As indicator of affective well-being, affect-balance (AB) score was computed by subtracting negative affect scores from positive affect scores. Cronbach’s-alpha at Q1-Q4 ranged from 0.83–0.88, indicating good internal reliability.

#### Knowledge (Q1, Q3).

Women’s knowledge on goals/limitations of the 13- and 20-week scans were evaluated by 10 and 8 items, respectively. Each questionnaire was divided into two sub-sections. The first, assessing knowledge on the goals of the scans, comprised 5 items (0–5 points). The second, evaluating knowledge on the limitations of the scans, comprised 5 (0–5 points) and 3 (0–3 points) items, respectively. For each correct answer, one point was allotted. Incorrect and absent/omitted answers were scored as zero. Knowledge for each 5-itemed sub-section was scored as “high” with at least 4/5 points, “moderate” with 3/5 and “low” with 2/5 or less points. The 3-itemed section on limitations of the 20-week scan was scored as “high” with 3/3 points, “moderate” with 2/3 and “low” with 1/3 points. Total knowledge scores were defined “high” when both sub-sections were scored as “high”, “moderate” when both sub-sections were scored as “moderate” or when one sub-section was scored as “moderate” and the other as “high”, and “low” when at least one of the two sub-sections was scored as “low”.

The questionnaire was designed ad-hoc by the researchers for the purpose of this study and reviewed and adjusted by a multidisciplinary expert panel, including fetal medicine specialists and a clinical psychologist with previous experience in constructing questionnaires.

#### Preferred timing (Q1,Q3).

Before undergoing the 13- and 20-week scans, women were asked to indicate their feelings towards the timing of the scans (“What do you think of the timing of this scan?”, response mode: 1 = “very late” to 5 = “very early”). They were also asked to express whether, and at which point during pregnancy, they wished to be informed about the presence/absence of fetal structural abnormalities (response mode: 1 = “as early as possible”, 2 =“during the 20 weeks scan”, 3 =“ I do not wish to be informed”).

#### Satisfaction and regrets (Q2, Q4).

Women were asked to state whether they had experienced feelings of regret and/or satisfaction following their decision to undergo the 13- and 20-week scans. The questionnaires were designed ad-hoc by the researchers and reviewed/adjusted by a multidisciplinary expert panel.

### Statistical analysis

We defined the following subgroups:

#### True-positives.

Abnormalities diagnosed at 13 weeks, confirmed after TOP/birth. False-positives: abnormalities diagnosed at 13 weeks, not confirmed later in pregnancy/post-partum. True-negatives: no abnormalities diagnosed at 13 weeks, 20 weeks and after birth.

Normally distributed variables were described by mean (SD), skewed distributions by median (range). Univariate analysis was performed for all measurement points (Q1-Q4). Unpaired-Student’s t-test and Mann-Whitney test were used to test for differences in continuous variables with normal or skewed distributions, respectively. Imputation of missing STAI/PANAS-items was performed by mean sum-score [14], when no more than 1/3 of items was missing at each measurement. Otherwise, sum/sub-scores were considered missing. Internal reliability of STAI, PANAS and affect-balance scores was evaluated using Cronbach’s-alpha coefficient. Differences in STAI- and PANAS-scores at Q1-Q2 and at Q3-Q4 were tested by Wilcoxon matched-pairs test for skewed distributions and paired Student’s t-test for normal distributions. Differences over time were evaluated by repeated measurements analysis (linear mixed model). Affect-Balance or STAI-scores were used as dependent variable at Q1 (baseline covariate). Time (Q2, Q3, Q4), educational level, results of the 13- and 20-week scans, and interaction effect between time and 13-week scan results were used as independent variables (covariance matrix: unstructured). All analyses were performed using SPSS version 23 (IBM-Corporation, NY-USA). All results were considered statistically significant when p<0.05 (two-sided).

### Ethics statement

For the study, a special license was obtained from the Ministry of Health, within the Dutch Population Screening Act11, regulating screening for incurable diseases [10]. The license number is 2014/31.

## Results

### Characteristics of participants

Fig 1 shows the study profile. During the questionnaire’s study period, 1512 women consented to participation. Response was 74% (n = 1118) at Q1, 64% (n = 941) at Q2, 55% (n = 807) at Q3 and 37% (n = 535) at Q4. Median time to complete each survey was 12 minutes (IQR 9–16 minutes). Of the 1500 (99%) women who underwent the 13-week scan a structural abnormality was diagnosed in 6 (0.4%) and confirmed after birth (or TOP in 2 cases). In 26 cases ultrasound markers (increased nuchal translucency, abnormal ductus venosus flow or tricuspid regurgitation) or other abnormalities (megacystis and intra-abdominal cyst) were diagnosed at 13 weeks but resolved or were not confirmed at later scans. Of the 1500 women, 1460 (96.5%) also underwent the 20-week scan and in 6 (0.4%) a structural abnormality was diagnosed at that time. Table 1 shows the maternal demographic and obstetric characteristics. Median maternal age was 33 years. Most women were highly educated (60.4%), married/in stable relationships (94.8%), employed (80.4%) and 76.4% nonreligious.

**Table 1:** Characteristics of participating pregnant women.

Characteristic (n = 1118)	n (%)
Maternal age (yr); median (range)	33 (18–49)
Educational level* (n = 1035)	
Low	68 (6.5)
Intermediate	336 (32.2)
High	631 (60.4)
Marital status (n = 1048)	
Married, stable relationship	993 (94.8)
Single	18 (1.7)
Others	37 (3.5)
Household status (n = 1050)	
Living with partner (and children)	993 (94.6)
Living with children	14 (1.3)
Living alone	26 (2.5)
Living with parents	5 (0.5)
Others	17 (1.6)
Working status (n = 1051)	
Employed	845 (80.4)
Unemployed	81 (7.7)
Student	13 (1.2)
Unfit to work	25 (2.4)
Other	87 (8.3)
Religious background (n = 1053)	
None	805 (76.4)
Christian	168 (16.0)
Muslim	10 (0.9)
Not sure	15 (1.4)
Others	55 (5.2)
**Obstetric history**	
Parity≥1 (n = 1110)	551 (49.6)
Termination of pregnancy for medical reasons (n = 1110)	26 (2.3)
History of miscarriage (n = 1118)	325 (29.6)

Numbers between brackets indicate the numbers of women who completed the question at Q1.

*Low: primary and lower secondary educational level

Intermediate: higher secondary education, medium vocational training

High: higher vocational training/college and university

### Maternal anxiety

**Fig 2:**
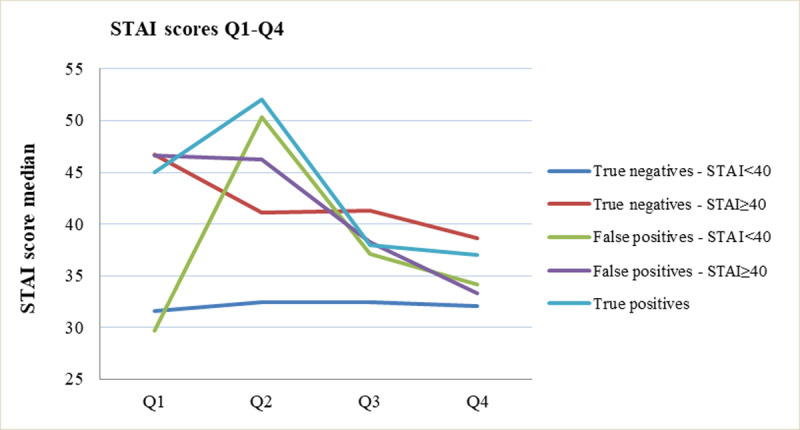
Median STAI scores before and after the 13-week and 20-week anomaly scans by subgroup. In women with true-negative results and STAI≥40 at Q1 (n = 312, 27.9%), anxiety was significantly lower after the 13-week scan (Q2: 40.0, 33.3–46.7 vs. Q1: 46.7, 40.0–50.0, p<0.01) and further decreased after the 20-week scan (Q4: 36.7: 33.3–43.3 vs Q3: 40.0: 36.7–46.7, p<0.01). In women with true-negative results and lower initial STAI-scores (STAI<40) (n = 707, 62.2%), no significant differences in median STAI-scores were found (Q2: 33.3, 30.0–36.7 vs Q1: 33.8, 26.6–36.7; p = 0.21 and Q4: 33.3, 26.7–36.7 vs Q3: 32.1, 24.6–35.2; p = 0.57).

Fig 2 shows mean (±SD) STAI-scores over time for responders with true-negative (n = 911, STAI-Q1 = 38.5± 9.7), true-positive (n = 4, STAI-Q1 = 45±12) and false-positive (n = 26, STAI-Q1 = 38.8±9.2) results at the 13-week scan. Of all women, 27.9% (312/1118) reported clinically relevant anxiety at Q1 (STAI≥40), 30.8% (290/941) at Q2, 25.8% (208/807) at Q3 and 26.6% (137/535) at Q4. For women with true-negatives at the 13-week scan, mean anxiety was lower after the scan (STAI Q2: 36.3±9.1 vs Q1: 38.5±9.7, p<0.01). Mean STAI-scores in this group were also lower after the 20-week scan (Q4: 35.1±9.2 vs Q3: 36.4±9.3, p<0.01). In true-positives at the 13-week scan, mean STAI-scores increased after the scan (STAI-Q2: 52.2±14.6 vs Q1: 45.0±12.3). In the four non-terminated pregnancies, anxiety levels decreased again after the 20-week scan (STAI-Q4: 37.4±4.7 vs Q3: 38.2±2.3). Similarly, in women with false-positives at 13 weeks, mean STAI-scores increased after the scan (Q2: 48.2±14.1 vs Q1: 38.9±9.9, p = 0.025), but decreased again after the 20-week scan (Q4: 33.8±4.2 vs Q3: 37.6±7.0, p = 0.36).

### Maternal affect

**Fig 3:**
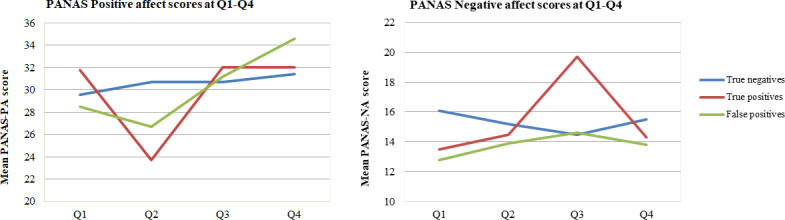
Individual well-being measured by mean PANAS-positive affect and PANAS-negative affect scores in the study period for all women. A higher PA score indicates a more favorable score. A higher NA score indicates a less favorable score.

Fig 3 shows mean PANAS-PA and PANAS-NA scores over time for women with true-negatives (n = 1468, Q1-PA: 29.6±7.1, NA: 16.2±5.5), true-positives (n = 4, Q1-PA: 31.7±1.5, NA: 15.0±3.1) and false-positives (n = 26, Q1-PA: 26.7±6.2, NA: 15±5.5). In women with true-negatives, PANAS-PA scores increased after the 13-week scan (Q2: 30.7±6.9 vs Q1: 29.1±7.2; p = 0.01) and after the 20-week scan (Q4: 31.4±8.2 vs Q3: 30.7±7.2, p = 0.04). The PANAS-NA scores significantly decreased after the 13-weeks scan (Q2: 15.2±5.1 vs Q1: 16.1±5.2, p<0.01), and remained stable after the 20-week scan (Q4: 15.5±5.2 vs Q3:14.5±4.8, p = 0.33). In women with true-positives at 13 weeks, PANAS-PA scores decreased after the scan (Q2: 23.5±7.6 vs Q1: 31.7±1.5). In the four true-positive cases that continued pregnancy, PA-scores remained stable after the 20-week scan (Q4: 32.0±1.4 vs Q3: 32.0±1.4). Mean PANAS-NA scores in the true-positive group increased after the 13-week scan (Q2: 14.5±0.7 vs Q1: 13.5±0.7) but decreased after the 20-week scan (Q4: 14.3±3.0 vs Q3: 19.7±3.8). In false-positive cases, the PANAS-PA scores decreased after the 13-week scan (Q2: 26.7±6.2 vs Q1: 28.5±8.4, p = 0.36) and increased again after the 20-week scan (Q4: 34.6±4.5 vs Q3: 31.2±5.9, p = 0.29). Mean PANAS-NA scores for false-positives were stable after the 13-week scan (Q2: 13.9±3.5 vs Q1: 12.8±2.3, p = 0.07) and decreased again after the 20-week scan (Q4: 13.8±4.3 vs Q3: 14.6±7.8, p = 0.20).

### Affect-balance

**Fig 4:**
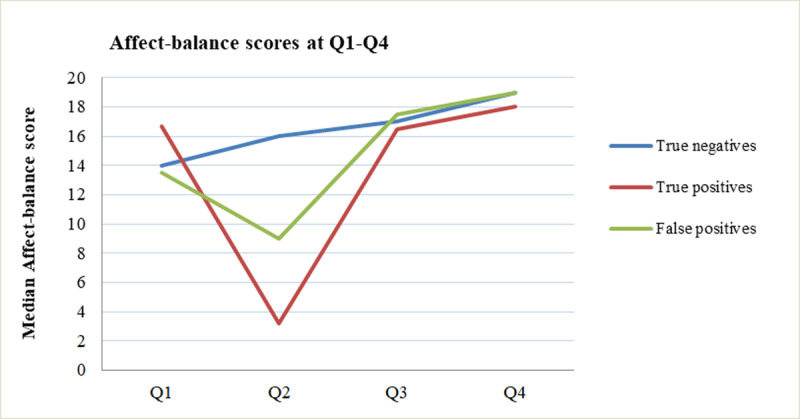
Median affect-balance scores before and after the 13-week and 20-week scans for true-negatives, true-positives and false-positives.

Fig 4 shows median (IQR) AB-scores over time for the three groups (Q1-AB true-negatives: 14.0, 7–21, true-positives: 17.0, 12.5–20.8, false-positives: 13.5, 2.7–22.3). In women with true-negatives, AB-scores constantly increased and reached the highest levels after the 20-week scan (Q2:16, 10.0–22.0 vs Q1:14, 7.0–21.0, p<0.01; and Q4:18, 10.0–24.0 vs Q3:17, 9.0–23.0, p = 0.006). In true-positives, median AB-scores decreased after the 13-week scan (Q2: -0.5:-3.5–13.0 vs Q1: 17.0,12.5–20.8) and normalized at the 20-week scan (Q4:18.0 vs Q3:15.0) in the four cases that decided to continue pregnancy. In women with false-positive results, median AB-scores decreased after the 13-week scan (Q2: -3.8–15.50 vs Q1:13.5, 2.8–22.3, p = 0.07) and increased again around 20 weeks (Q4:19.0, 17.0–26.0 vs Q3:17.5, 12.0–20.0, p = 0.28).

S1 Table shows the results of the multivariable analysis of STAI- and AB-scores over time. STAI-score was significantly higher at Q2 and Q3 than at Q4. AB-scores improved significantly over time compared to Q4, regardless of educational level or 20-week scan results. Finally, the profile of STAI- and AB-scores over time was positively associated with the respective baseline scores at Q1.

Table 2 shows women’s feelings of satisfaction/ regrets about their decisions of undergoing the 13-week scan. About 80% (n = 692) of women were very-to-extremely satisfied to have undergone the 13-week scan and 97.1% (n = 825) stated they did not regret this at all.

**Table 2:** Feelings of satisfaction and regret of pregnant women regarding their decision to undertake the 13-week anomaly scan (Q2).

Question	n (%)				
	Not at all	A little	Quite	Very	Extremely
*I felt relieved by the findings of the 13-week anomaly scan (n = 847)*	31 (3.7)	68 (8.0)	147 (17.3)	408 (48.2)	193 (22.8)
*I was disappointed with the findings of the 13-week anomaly scan (n = 845)*	795 (94.1)	26 (3.1)	8 (1.0)	8 (1.0)	8 (1.0)
*I regret having undergone the 13-week anomaly scan (n = 850)*	825 (97.1)	11 (1.3)	4 (0.5)	3 (0.4)	7 (0.8)
*I am satisfied with my decision (n = 848)*	30 (3.5)	19 (2.2)	107 (12.6)	427 (50.4)	265 (31.3)
*I was happy I had chosen for the 13-week anomaly scan (n = 846)*	16 (1.9)	28 (3.3)	80 (9.5)	394 (46.6)	328 (38.8)
*I had not expected this result at all (n = 846)*	784 (92.7)	29 (3.4)	10 (1.2)	16 (1.9)	7 (0.8)
*I was reassured by the findings of the 13-week anomaly scan (n = 844)*	52 (6.2)	64 (7.6)	144 (17.1)	389 (46.1)	195 (23.1)
*I became insecure after the findings of the 13-week scan (n = 850)*	595 (70.0)	197 (23.2)	32 (3.8)	17 (2.0)	9 (1.1)

#### Knowledge (Q1, Q3).

Table 3 shows that 87.1 (= 33.9% moderate + 53.2% high) % and 88.6% (= 38.7% moderate + 49.9% high) of women obtained moderate-to-high total scores on the goals and limitations of the 13- and 20-week scans, respectively. S2 Table shows respondents’ knowledge item scores. Knowledge on the goals of the 13-week scan was low in 11.2% (n = 117) of women, while knowledge on the limitations of the scan was low in 2.4% (n = 25). Similarly, knowledge on the goals of the 20-week scan was low in 6.8% (n = 42) of women, versus 6.0% (n = 37) for the limitations of the scan.

**Table 3:** Distribution of the level of knowledge on goals and limitation of the 13- and the 20-week scans.

Level of knowledge	n (%)					
	13-week scan (Q1) (n = 1045)			20-week scan (Q3) (n = 615)		
	Total	Goals	Limitations	Total	Goals	Limitations^#^
Low	135 (12.9)	117 (11.2)	25 (2.4)	70 (11.4)	42 (6.8)	37 (6.0)
Moderate	354 (33.9)	313 (29.9)	87 (8.3)	238 (38.7)	116 (18.9)	179 (29.1)
High	556 (53.2)	615 (58.9)	933 (89.3)	307 (49.9)	457 (74.3)	399 (64.9)

### Preferences

Table 4 shows women’s preferences for the timing of the scans in pregnancy. The majority (n = 816, 76.4%) found the timing of the 13-week scan ‘not late, nor early’. A total of 506 (81%) women found the timing of the 20-week scan ‘not late nor early’ but 109 women (17.3%) judged it as “late”. Almost all women stated, at Q1 (99.0%) and Q3 (97.0%), that they wished to be informed as early as possible in pregnancy about fetal structural abnormalities.

**Table 4:** Preferred timing of screening by pregnant women for fetal structural abnormalities.

Question	13 week scan (n = 1068)			20 week scan (n = 627)		
		n	%		n	%
*What do you think of the timing of this scan*?	*-Really very late*	0	0	*-Really very late*	2	0.3
	*-Late*	18	1.7	*-Late*	109	17.3
	*-Not late*, *not early*	816	76.4	*-Not late*, *not early*	506	81.1
	*-Early*	224	21.0	*-Early*	9	1.3
	*-Really early*	10	0.9	*-Really early*	0	0
*If during an ultrasound scan an abnormality is found in the organs of your baby*, *when would you wish to be informed*?	*-As early as possible*	1058	99.0	*-As early as possible*	608	97.0
	*-During the 20-week scan*	10	0.9	*-During the 20-week scan*	17	2.7
	*-I do not wish to be informed*	2	0.2	*-I do not wish to be informed*	2	0.3

## Discussion

### Main findings

Our study shows that undergoing the 13-week anomaly scan did not negatively affect maternal psychological outcomes. We found a decrease in anxiety levels and an increase in well-being in mothers with normal findings at the early scan. In women with false positive results, well-being and anxiety levels temporary increased following false-positive results at the early scan but normalized again once the abnormality was disproved. The majority of women (87.1%) obtained moderate-to-high total scores on the knowledge items of the 13- and 20-week scans and virtually all women (99%) wished to be informed as early as possible during pregnancy about structural abnormalities.

### Interpretation

In the current study, 27.9% of women had clinically relevant anxiety levels before the 13-week anomaly scan. Clinically relevant state-anxiety has been reported in 20.5–25.2% of women during pregnancy, and more specifically in 19.1% during the first trimester [15]. The higher prevalence in our study may indicate that our Q1 measurements cannot be considered representative of true baseline. As the questionnaires were filled-in shortly before the 13-week scan, higher anxiety levels may be due to maternal anticipatory anxiety in view of the imminent scan. However, in women with clinically relevant STAI-scores at Q1, the decrease in anxiety levels after the 13-week scan was twice as great as after the 20-week scan. We therefore hypothesize that a normal 13-week scan might be more effective than a later scan at reassuring “anxious” pregnant women. However, we were unable to compare our findings due to the lack of literature on the impact of a 20-week anomaly scan on maternal anxiety. Our study confirms previous findings on early screening for Down Syndrome, when an ultrasound examination at 11–13+6 weeks did not increase maternal anxiety levels at 20 weeks [16, 17], even in case of false-positive results [18, 19] An argument in favor of postponing fetal anatomical assessment to the second trimester of pregnancy is the potential increase in parental anxiety after false-positive results [20]. However, the false-positive rate for structural abnormalities at the 13-week anomaly scan has been reported to be a rare occurrence (0.1–0.3%) [2, 3]. Even though we found a temporary increase in anxiety levels and decrease in well-being following false-positive results at the early scan, these normalized again in the course of pregnancy indicating no long-term detrimental effect on maternal anxiety and well-being. Moreover, despite the small number of cases, our results are consistent with previous literature on pregnancies with true-positive findings. In agreement with Kaasen et al [21], women carrying fetuses with known abnormalities initially experience high stress scores, which gradually decrease to equal those of women with a normal fetus. We also confirm that in women with uncomplicated pregnancies psychological-stress levels are constantly low during the latter half of gestation.

The majority of women (87.1%) obtained moderate-to-high total scores on knowledge items on the 13- and 20-week scans. The finding that Dutch women are adequately informed about prenatal screening has been previously reported [22], but other studies point to considerable variability among regions and ultrasound practices [23–25]. As women in our study were aware that their responses were being recorded, a bias by Hawthorne effect cannot be excluded. Contrary to previous reports [23], educational level did not significantly affect the answers of participants. However, only 6.5% of women in our study had a low educational level. Although most women scored moderate or high on knowledge items, 16% of them believed that the 13-week scan could entirely replace the 20-week scan, indicating the need for further improvements in prenatal counseling. To maximize efficacy of counseling, additional tools like group education [26] and (electronical) decision aids [27–29] have been proposed.

The majority of severe/lethal fetal abnormalities are detectable in the first trimester of pregnancy [2, 5, 6, 30]. In the 90s, women had already expressed a strong preference for early screening for Down Syndrome [31–33]. However, up to now, little was known about their preference regarding early screening for structural abnormalities. Our results show that women clearly favor early screening for structural abnormalities, even in case of abnormal findings. Ninety-nine percent of women wished to be informed about fetal structural abnormalities as early as possible in pregnancy and up to 97% of them confirmed this when asked again at the time of the 20-week scan. Similarly, Maiz et al. showed that 94% of women wish to know about fetal structural abnormalities in the first-trimester of pregnancy, regardless of the severity of the abnormality [34].

### Strengths and limitations

To our knowledge, this is the first study to longitudinally investigate maternal psychological outcomes following a 13-week scan and using validated questionnaires. Even though our sample size was large, one study limitation is the drop in response rates from 74% (Q1) to 37% (Q4). While demographic and obstetric characteristics between responders and drop-outs did not significantly differ, a selection bias through unmeasured/omitted variables cannot be excluded. Furthermore, we are unable to report on women who declined the 13-week scan as we did not follow-up on those cases. Although the inter-case variability of women with screen-positive results in our cohort was high, the number and effect sizes were too small to reach statistical significance. Also, we cannot report on women with false-negative results at the 13-week scan as we had none in our cohort^2^. Finally, it is noteworthy that women with false-positives were slightly over-represented among responders.

## Conclusion

The 13-week anomaly scan did not negatively affect the psychological well-being of pregnant women with screen-negative test results. The small number of women with screen-positive results temporarily experienced higher anxiety levels after the scan but, in case of false-positives, as we expected, these normalized when the abnormality was not confirmed at follow-up. In the long term, psychological outcomes of these women did not differ from those of screen-negative women. Future studies with larger cohorts are needed to confirm these findings. Finally, most pregnant women scored moderate-to-high on knowledge of the goals and limitations of the 13-week scan and strongly prefer early screening for fetal structural abnormalities.

## Supporting information

S1 Table Repeated measurements analysis for STAI, Affect-balance scores over time. (DOCX)

S2 Table Answers of women to questions on scope and limitation of the 13 and the 20-week scans. (DOCX)

S1 File Questionnaires used–dutch version. (DOCX)

S2 File Questionnaires used–english version. (DOCX)
