# Supplementary material for: Psychological outcomes, knowledge and preferences of pregnant women on first-trimester screening for fetal structural abnormalities: A prospective cohort study
Source: PLoS One. 2021 Jan 27;16(1):e0245938. doi: 10.1371/journal.pone.0245938 (PMC7840026; doi:10.1371/journal.pone.0245938)
Supplement: S1 Table — (DOCX) [file pone.0245938.s001.docx]

**S1 Table. Repeated measurements analysis* for STAI, Affect-balance scores over time.**

| Variable | STAI score B-coefficient (95% CI) |  | PANAS score B-coefficient (95% CI) |  |
| --- | --- | --- | --- | --- |
|  |  | P |  | P |
| Constant | 31.6 (17.5; 45.7) | <.001 | 62.5 (29.4- 95.6) | <.001 |
| Time  Q2, after 13wk scan  Q3, before 20wk scan  Q4, after 20wk scan (ref) | 12.1 (1.2; 22.9)  1.4 (0.6; 2.2)  0 | .029 <.001  ̶ | -14.4 (-39.6; 10.9) - 3.1 (- 4.9; - 1.3)  0 | .27 .001  ̶ |
| Result 13wk scan  negative  false-positive  true-positive (ref) | -4.1 (-13.9; 5.8) -4.0 (-14.5; 6.5)  0 | .42 .46  ̶ | -7.4 (-29.6; 14.8) -7.3 (-31.1; 16.4)  0 | .51 .55  ̶ |
| Interaction effect  Time Q2 x negative 13wk scan  Time Q2 x false-positive 13wk scan | -10.9 (-21.7; -0.1)  0.3 (-11.3; 11.8) | .049 .97 | 11.9 (-13.4; 37.1) 15.8 (-11.2; 42.7) | .36 .25 |
| Result 20 weeks scan  negative  positive (ref) | 3.9 (-6.2; 14.1) 0 | .44  ̶ | 11.0 (-12.6; 34.6)  0 | .36  ̶ |
| Educational level Not reported   low  middle  high (ref) | -3.8 (15.0; 7.4)  2.2 (- 0.0; 4.3)  0.2 (- 0.8; 1.3)  0 | .50  .05  .65  ̶ | -10.8 (-36.0; 14.4)  3.4 (- 1.4; 8.2)  0.8 (- 1.7; 3.2)  0 | .40 .17 .55  ̶ |
| STAI at Q1  <40 (ref)   ≥40 | 0 7.8 (6.8; 8.8) | ̶ <.001 | n.a. n.a. | n.a. n.a. |
| Affect-balance Q1  (continuous score) | n.a. | n.a. | 0.59 (0.54; 0.63) | <.001 |
|  |  |  |  |  |
|  |  |  |  |  |

*Linear mixed model; AIC goodness of fit measure 12932.4 (STAI), 12138.1 (Affect Balance)
ref: reference category
