# Supplementary material for: Psychological outcomes, knowledge and preferences of pregnant women on first-trimester screening for fetal structural abnormalities: A prospective cohort study
Source: PLoS One. 2021 Jan 27;16(1):e0245938. doi: 10.1371/journal.pone.0245938 (PMC7840026; doi:10.1371/journal.pone.0245938)
Supplement: S2 Table — (DOCX) [file pone.0245938.s002.docx]

S2 Table. Answers of women to questions on scope and limitation of the 13 and the 20-week scans.

|  | 13 week scan (Q1) | | | |  | 20 week scan (Q3) | | | | |
| --- | --- | --- | --- | --- | --- | --- | --- | --- | --- | --- |
|  | **True** |  | **False** | | | **True** | |  | **False** | |
| *The goal of the 13 week scan is to* | n/total | % | n/total | % | | n/total | **%** | | n/total | % |
| 1. *Check whether the organs of your baby are abnormal* | 974/1077 | 90.4 | 103/1077 | 9.6 | | 619/633 | 97.8 | | 14/633 | 2.2 |
| 1. *Check whether your baby has chromosomal abnormalities* | 620/1077 | 57.6 | 457/1077 | 42.4 | | 211/633 | 33.3 | | 422/633 | 66.7 |
| 1. *Check whether your baby has developmental abnormalities* | 312/1077 | 29.0 | 765/1077 | 7.1 | | 118/633 | 18.6 | | 515/633 | 81.4 |
| 1. *Check if the heart of your baby is beating* | 274/1077 | 25.4 | 803/1077 | 74.6 | | 213/633 | 33.7 | | 420/633 | 66.4 |
| 1. *To calculate the estimated date of delivery* | 94/1077 | 8.7 | 983/1077 | 91.3 | | 34/633 | 5.4 | | 599/633 | 94.6 |
| 1. *During the scan not all structural abnormalities can be seen because some organs have not yet sufficiently developed* | 991/1065 | 93.0 | 74/1065 | 7.0 | | 417/629 | 66.3 | | 212/629 | 33.7 |
| 1. *If during the scan no structural abnormalities are seen, this means that my baby will be healthy* | 73/1067 | 6.8 | 994/1067 | 93.2 | | 34/627 | 5.4 | | 593/627 | 94.6 |
| 1. *During the scan an abnormality with a yet unclear significance for the baby might be seen* | 1029/1065 | 96.6 | 36/1065 | 3.3 | | 614/629 | 97.6 | | 15/629 | 2.4 |
| 1. *If a 13 week scan does not show any abnormality, this means that my baby does not have Down Syndrome* | 122/1068 | 11.4 | 946/1068 | 88.6 | |  | | | | |
| 1. *The 13 week scan can replace the 20 week scan* | 172/1071 | 16.0 | 899/1071 | 84.0 | |  | | | | |
