# Supplementary material for: Psychological outcomes, knowledge and preferences of pregnant women on first-trimester screening for fetal structural abnormalities: A prospective cohort study
Source: PLoS One. 2021 Jan 27;16(1):e0245938. doi: 10.1371/journal.pone.0245938 (PMC7840026; doi:10.1371/journal.pone.0245938)
Supplement: S1 File — (DOCX) [file pone.0245938.s003.docx]

**S1 File.**

**Questionnaires used – Dutch version**

**Anxiety by STAI scale (used at Q1-Q4)**

Hieronder vindt u een aantal uitspraken, welke mensen gebruiken om zichzelf te beschrijven. Lees iedere uitspraak door en omcirkel het cijfer rechts van die uitspraak om daarmee aan te geven hoe u zich de AFGELOPEN dagen heeft gevoeld.

- Ik voel me kalm
- Ik ben gespannen
- Ik ben in de war
- Ik ben ontspannen
- Ik voel me tevreden
- Ik maak me zorgen

Likert scale:

1. Geheel niet
2. Een beetje
3. Tamelijk veel
4. Zeer veel

**Affect by PANAS scale (used at Q1-Q4)**

Hieronder ziet u een aantal woorden welke verschillende gevoelens en emoties beschrijven. Wilt u bij ieder woord aangeven in welke mate u zich de AFGELOPEN dagen zo heeft gevoeld door het antwoord te omcirkelen dat op u van toepassing is.

- Aandachtig
- Vijandig
- Geïnteresseerd
- Prikkelbaar
- Alert
- Schuldig
- Uitgelaten
- Beschaamd
- Enthousiast
- Nerveus
- Geïnspireerd
- Rusteloos
- Trots
- Overstuur
- Vastberaden
- Van streek
- Sterk
- Bang
- Actief
- Angstig

Likert scale:

- Nauwelijks of helemaal niet
- Een beetje
- Tamelijk
- Vrij sterk
- In zeer hoge mate

**Knowledge – developed by the researchers**

**Questionnaire used at Q1:**

Onderstaande vragen gaan over de 13 Weken Echo.

Het doel van de 13 weken echo is (u mag meerdere mogelijkheden aankruisen):

- Het opsporen van lichamelijke afwijkingen
- Het opsporen van geestelijke afwijkingen
- Het opsporen van erfelijke (chromosomale) afwijkingen
- Of het hartje van mijn baby nog klopt
- Om de zwangerschapsduur van mijn zwangerschap te bepalen

U mag nu aangeven of het antwoord juist of onjuist is:

- Bij de 13 weken echo kunnen nog niet alle afwijkingen gezien worden omdat sommige organen nog niet goed genoeg ontwikkeld zijn.
- Wanneer de 13 weken echo geen afwijkingen laat zien, wil dat zeggen dat mijn kind gezond is.
- Bij de 13 weken echo kan een afwijking worden gezien waarvan op dat moment nog niet duidelijk is wat de betekenis daarvan is.
- Wanneer de 13 weken echo geen afwijkingen laat zien, wil dat zeggen dat mijn kind geen Down syndroom heeft.
- De 13 weken echo kan de 20 weken echo vervangen

**Preferred timing (used at Q1,Q3) – developed by the researchers**

Wat vindt u van het tijdstip in de zwangerschap waarop de 13 weken echo plaatsvindt?

- Zeer laat
- Laat
- Niet laat, niet vroeg
- Vroeg
- Zeer vroeg

Als tijdens een echo-onderzoek blijkt dat uw kindje een lichamelijke afwijking heeft, wanneer zou u dat dan willen weten?

- Zo snel mogelijk
- Bij de twintig weken echo (SEO)
- Ik wil dat niet weten

**Satisfaction and regrets (used at Q2, Q4) – developed by the researchers**

- Ik voelde me opgelucht door de uitslag
- Ik voelde me teleurgesteld door de uitslag
- Ik had spijt dat ik voor de echo had gekozen
- Ik voelde me tevreden
- Ik was blij dat ik voor de echo had gekozen
- Ik schrok van de uitslag
- Ik was gerustgesteld door de uitslag
- Ik werd/bleef onzeker door de uitslag

Likert scale answers:

- Nauwelijks of helemaal niet
- Een beetje
- Tamelijk
- Vrij sterk
- In zeer hoge mate
