# Supplementary material for: Psychological outcomes, knowledge and preferences of pregnant women on first-trimester screening for fetal structural abnormalities: A prospective cohort study
Source: PLoS One. 2021 Jan 27;16(1):e0245938. doi: 10.1371/journal.pone.0245938 (PMC7840026; doi:10.1371/journal.pone.0245938)
Supplement: S2 File — (DOCX) [file pone.0245938.s004.docx]

**S2 File.**

**Questionnaires used – English version**

**Anxiety by STAI scale (used at Q1-Q4)**

How do you feel right now?

- I feel calm
- I am tense
- I feel upset
- I am relaxed
- I am content
- I am worried

Likert scale answers:

1. Not at all
2. Somewhat
3. Moderately
4. Very much

**Affect by PANAS scale (used at Q1-Q4)**

Indicate the extent you have felt this way over the past week.

- Attentive
- Hostile
- Interested
- Irritable
- Alert
- Guilty
- Excited
- Ashamed
- Enthusiastic
- Jittery
- Inspired
- Distressed
- Proud
- Upset
- Determined
- Upset
- Strong
- Scared
- Active
- Nervous

Likert scale answers:

- Very slightly or not at all
- A little
- Moderately
- Quite a bit
- Extremely

**Knowledge – developed by the researchers**

**Questionnaire used at Q1:**

Please indicate whether the following statements are true or false.

The goal of the scan^§^ is to:

- Check whether the organs of your baby are abnormal
- Check whether your baby has developmental abnormalities
- Check whether your baby has chromosomal abnormalities
- Check if the heart of your baby is beating
- Calculate the estimated date of delivery

Please indicate whether the following statements are true or false:

- During the 13-week scan not all structural abnormalities can be seen because some organs have not yet sufficiently developed
- If during the scan no structural abnormalities are seen, this means that my baby will be healthy
- During the scan an abnormality with a yet unclear significance for the baby might be seen
- If a 13-week scan does not show any abnormality, this means that my baby does not have Down Syndrome
- The 13 week-scan can replace the 20 week scan

§ we asked about the 13-week scan at Q1 and about the 20-week scan at Q3

**Preferred timing (used at Q1,Q3) – developed by the researchers**

What do you think of the timing of this scan?

- Very late
- Late
- Not too late, not too early
- Early
- Very early

If during an ultrasound scan an abnormality is found in the organs of your baby, when would you wish to be informed?

- As early as possible
- During the 20-week scan
- I do not wish to be informed

**Satisfaction and regrets (used at Q2, Q4) – developed by the researchers**

- I felt relieved by the findings of the scan
- I was disappointed with the findings of the scan
- I regret having undergone the scan
- I am satisfied with my decision
- I was happy I had chosen for the scan
- I had not expected this result at all
- I was reassured by the findings of the scan
- I became insecure after the findings of the scan

Likert scale answers:

- Very slightly or not at all
- A little
- Moderately
- Quite a bit
- Extremely
